# Superoxide Anion Production and Bioenergetic Profile in Young and Elderly Human Primary Myoblasts

**DOI:** 10.1155/2018/2615372

**Published:** 2018-07-24

**Authors:** Mariangela Marrone, Rita Maria Laura La Rovere, Simone Guarnieri, Ester Sara Di Filippo, Giovanni Monaco, Tiziana Pietrangelo, Geert Bultynck, Stefania Fulle, Rosa Mancinelli

**Affiliations:** ^1^Department of Neuroscience Imaging and Clinical Sciences, University “G. d'Annunzio” Chieti-Pescara, Via dei Vestini 29, 66100 Chieti, Italy; ^2^Interuniversity Institute of Myology (IIM), Chieti, Italy; ^3^Laboratory of Molecular and Cellular Signaling, KU Leuven, Campus Gasthuisberg O/N-I bus 802, Herestraat 49, 3000 Leuven, Belgium

## Abstract

Sarcopenia is the age-related loss of skeletal muscle mass, strength, and function. It is associated with regenerative difficulties by satellite cells, adult muscle stem cells, and alteration of oxidative management, mainly the increase in superoxide anions (O_2_^•−^). We aimed to investigate the relation between regenerative deficit in elderly and increase in O_2_^•−^ production along with mitochondrial alterations. Myoblasts and myotubes from skeletal muscle of young and elderly healthy subjects (27.8 ± 6 and 72.4 ± 6.5 years old) were measured: (1) superoxide dismutase activity and protein content, (2) mitochondrial O_2_^•−^ production levels, (3) O_2_^•−^ production variability, and (4) mitochondrial bioenergetic profile. Compared to young myoblasts, elderly myoblasts displayed decreased SOD2 protein expression, elevated mitochondrial O_2_^•−^ baseline levels, and decreased oxidative phosphorylation and glycolysis. Additionally, elderly versus young myotubes showed elevated mitochondrial O_2_^•−^ levels when stressed with N-acetyl cysteine or high glucose and higher glycolysis despite showing comparable oxidative phosphorylation levels. Altogether, the elderly may have less metabolic plasticity due to the impaired mitochondrial function caused by O_2_^•−^. However, the increased energy demand related to the differentiation process appears to activate compensatory mechanisms for the partial mitochondrial dysfunction.

## 1. Introduction

Sarcopenia is defined as the age-related loss of muscle mass, strength, and function [1]. In particular, the aging process is associated with a consistent decrease in the ability of muscle tissue to regenerate following injury or overuse due to the impairment of intervening satellite cells [2]. In response to muscle damage, satellite cells, undifferentiated quiescent mononucleated cells present in muscle [3], which have properties of stem cells, are activated to proliferate as human primary myoblasts Proliferating myoblasts migrate to the damaged region of the fiber, where they differentiate and fuse to form myotubes via a similar process to that of myogenesis [4]. However, this capacity is reduced in the elderly, where satellite cells are unable to execute the complete repair process or they exhibit a slow recovery [5–7]. Activation and proliferation stages of satellite cells are characterized by the expression of myogenic regulatory factors (MRFs), and the phases of this process typically involve the sequential expression of proteins, including Pax3 (quiescent satellite cells), myoD (proliferating primary myoblasts), and myogenin (differentiated myoblasts). We previously demonstrated that the impaired regenerative potential in elderly primary myoblasts is related to dysregulation in myogenin and miR-1 and 133b gene expression together with an overproduction of reactive oxygen species (ROS) [8]. Indeed, the mechanisms behind the impaired myogenic differentiation process of elderly people are multifactorial and they appear to involve an imbalance between ROS production and antioxidant enzyme activity. Thus, oxidative stress can be generated in the cells as a result of one or more of three factors: (i) increase in oxidant generation, (ii) decrease in antioxidant protection, and (iii) failure to repair oxidative damage. ROS cause damage to lipids, nucleic acids, and proteins [9]. Mitochondria are commonly considered as the source and target of ROS in many cell models including skeletal muscle cells. Noteworthily, as myoblast differentiation is accompanied by intensive mitochondrial biogenesis, the generation of intracellular ROS is also increased during myogenesis [10], thus playing a fundamental role in aging. Aging is associated with a decline in mitochondrial function and the accumulation of abnormal mitochondria [11]. Indeed, several studies demonstrate that a link exists between increased mitochondrial dysfunction and increased ROS production in sarcopenic skeletal muscle [12]. The electron transfer for complete reduction of oxygen by mitochondrial complexes I and III–IV could be the main source of ROS, and specifically O_2_^•−^ [13]. The major class of enzymatic antioxidants is the superoxide dismutase (SOD) family that catalyze the dismutation of O_2_^•−^ to hydrogen peroxide (H_2_O_2_) [14]. Subsequently, H_2_O_2_ is quickly reduced to water by two other enzymes, catalase and glutathione peroxidase. The isoforms of SOD are different in their cellular location: SOD1 is located in cytosol and mitochondrial intermembrane space, SOD2 in mitochondrial matrix, and SOD3 in extracellular space. Mitochondria are also the “powerhouse” of the cells producing energy in the form of adenosine triphosphate (ATP) [15, 16]. In eukaryotes, ATP is mainly produced by two different processes: through the breakdown of glucose or other sugars in the cytosol (anaerobic glycolysis) or by the metabolism of fatty acids, sugars (aerobic glycolysis), and proteins in the mitochondria, solely in the presence of oxygen (oxidative phosphorylation (OXPHOS)). The glycolytic pathway converts glucose to pyruvate in an oxygen-independent biochemical reaction resulting in ATP production. One fate of the pyruvate is reduction to lactate in the cytosol with net proton production, acidifying the medium. Protons are pumped out of the cell by various mechanisms to maintain the intracellular pH [17], and the efflux of the protons into the extracellular space or medium surrounding the cells causes extracellular acidification [18, 19]. The major nutrient substrates glucose, glutamine, and fatty acids can be completely oxidized into CO_2_ and H_2_O via the mitochondrial tricarboxylic acid cycle (TCA) and associated OXPHOS. OXPHOS takes places through the respiratory chain, namely, complexes I–V, that transfers electrons obtained from TCA NADH or FADH_2_ across the mitochondrial inner membrane and the terminal electron acceptor, oxygen (O_2_). More in detail, the electron transport chain passes a high energy electron at the inner mitochondrial membrane pumping hydrogen out of the matrix space, creating a proton motive force (Δp) across the inner membrane with both electrical (Δ*Ψ*m) and chemical (ΔpH) components [20]. The gradient created drives hydrogen back through the membrane, through ATP synthase that couples it to ATP production by OXPHOS. The CO_2_ produced can be converted to bicarbonate and protons as catalyzed by carbonic anhydrase [17], another source of protons causing medium acidification. The OXPHOS is a process through which ADP is phosphorylated in ATP by the energy derived from the oxidation of nutrients in aerobic condition. It takes places through the respiratory chain, named complexes I–V conducting electron transfer across the mitochondrial inner membrane.

Although the major part of oxygen is used for ATP synthesis (coupled respiration), the mitochondrial membrane is leaky and protons may leak back through the membrane, increasing oxygen consumption (uncoupled respiration). There are three leak events: complex I leaks O_2_^•−^ towards the mitochondrial matrix, while complex III leaks O_2_^•−^ towards both the intermembrane space and the mitochondrial matrix [21, 22].

We hypothesized that the deficit in regeneration in elderly could be related to the increased O_2_^•−^ production and mitochondrial alterations. To verify this, primary myoblasts were isolated from human skeletal muscle of young and aged healthy subjects and measured as undifferentiated and differentiated cells: (1) SOD activity and protein content, (2) mitochondrial O_2_^•−^ production, (3) variability in O_2_^•−^ production, and (4) mitochondrial bioenergetics profile. The data from this study suggest that in elderly primary myoblasts, the metabolic machinery may not work properly. The analysis of the bioenergetic profile in elderly myoblasts seems to suggest a defect in mitochondrial function and a different metabolic plasticity with respect to young ones. Although elderly and young myotubes showed a similar oxidative metabolism, surprisingly in elderly myotubes, we found an increased level of mitochondrial superoxide anion relying more on the glycolysis processes. Taken together, these findings would suggest a glycolytic compensation in response to mitochondrial oxidative damage.

## 2. Materials and Methods

### 2.1. Muscle Samples

Biopsies from vastus lateralis muscles were obtained from 13 male healthy untrained subjects who voluntarily participated in the study. Based on age, the subjects were divided into two experimental groups:

1. Young subjects, 27.8 ± 6 years old (*n* = 8)
2. Elderly subjects, 72.4 ± 6.5 years old (*n* = 5)

Biopsies were performed following the procedure described in Pietrangelo et al. [23] and treated to collect satellite cell populations as previously described in Fulle et al. [9]. All of the subjects provided written, informed consent before participating in the study. The study was conducted according to the Helsinki Declaration (as amended in 2000), and it was approved by Ethic Committee of “G. d'Annunzio” University of Chieti-Pescara, Italy (PROT 1233/06, 1634/08, 1884/09 COET). The inclusion criteria were normal ECG and blood pressure and absence of metabolic, cardiovascular, chronic bone/joint, and muscular diseases. Exclusion criteria were the presence of metabolic and/or cardiovascular diseases, evidence of hereditary or acquired muscular disease, or diagnosis of respiratory or psychiatric disorders. No subject was under treatment with testosterone or other pharmacological interventions or training protocols known to influence skeletal muscle. All subjects were Caucasian and nonsmokers. They presented BMI (body mass index) and % FM (fat mass) values that were not statistically different (BMI: 24.2 ± 1.5 and 27.6 ± 1.7, young and elderly, resp.; % FM 23.0 ± 2.1 and 26.3 ± 2.1 young and elderly, resp.).

### 2.2. Satellite Cell Populations

The satellite cells were obtained, expanded as myoblasts in growth medium, and differentiated as previously described [9, 24]. Briefly, the percentages of myogenicity of the cell cultures were obtained using an immunocytochemistry assay, with the marker desmin and with biotinylated streptavidin-AP kits (Dako REAL™ Detection System Peroxidase/DAB+, Rabbit/Mouse; cat. number K5001; DAKO, DakoCytomation, Glostrup) [25]. Desmin is a cytoskeleton intermediate filament protein expressed early in myogenic populations [26, 27]. Differentiation of the cell populations was determined by counting the number of nuclei in the myotubes after 7 days of differentiation, as percentages with respect to the total number of nuclei, with the ratio between these two values (nuclei in myotubes/total nuclei × 100%) giving the fusion index. We only considered myotubes that were positive to the primary antibody against myosin heavy chain (MHC), using the MF20 anti-MHC monoclonal antibody (diluted 1 : 50; Developmental Studies Hybridoma Bank, University of Iowa, Iowa City, IA, USA), and that contained three or more nuclei [6].

### 2.3. Fluorescence-Activated Cell Sorting

Myogenic precursors expressing both CD56 and 5.1H11 were isolated by fluorescence-activated cell sorting (FACS) at FACSAria III (BD) as described in di Filippo et al. [8]. The primary antibodies used for this technique are specific for human myoblasts that targeted a cell surface protein, as CD56 and 5.1H11. These human CD56^+^/5.1H11^+^ cells were replaced in petri dishes and cultured in growth or differentiation medium for further analysis.

### 2.4. Ca^2+^ Imaging and Videomicroscopy Experiments

Videomicroscopy experiments are performed on cells plated on the bottom of special 96-well plates at a confluence of 10,000 cells/cm^2^. The loading procedure is previously described by di Filippo et al. [28]. After registration of baseline, 40 mM KCl was added to the cells and the registration was continued [29]. The living cells, loaded with Fura-2, were sequentially excited at 340 or 380 nm with a high-speed wavelength switcher Polychrome II (Till Photonics, Germany). Fluorescence images were collected using a 40x oil objective lens, acquired using an intensified CCD camera (Hamamatsu Photonics, Hamamatsu, Japan), stored on a PC, and analyzed off-line. The acquisition time for each fluorescence emission was 0.5 s. It has been taken into consideration the ratio fluorescence (340/380 nm) signal in a selected representative cellular area after the subtraction of background fluorescence.

### 2.5. Protein Isolation and Quantitation

Antioxidant enzyme assays and Western blotting were performed using myoblasts and myotubes lysed in RIPA buffer (number R0278, Sigma-Aldrich), supplemented with 1 : 100 protease and phosphatase inhibitor cocktail (number P8340, Sigma-Aldrich), incubated 1 hour at 4°C, and centrifuged at 10000 rpm for 10 minutes at 4°C. Cytosol protein concentrations were measured on the resulting supernatant according to Bradford's method (Bradford Reagent, number B6916, Sigma-Aldrich).

### 2.6. Superoxide Enzyme Activities

Superoxide dismutase (SOD) activity was determined using the modified method of L'Abbé and Fischer [30, 31]. The final assay volume (1 ml) contained 20 mM Na_2_CO_3_, pH 10, 10 mM cytochrome c (number C7752, Sigma Aldrich), and 1 mM xanthine (number X0626, Sigma-Aldrich) and xanthine oxidase (number X4500, Sigma-Aldrich). As the xanthine oxidase activity varies, the amount used for the assay was sufficient to stimulate cytochrome c reduction at 550 nm at a rate of 0.025 per minute without SOD addition. SOD units were calculated on the basis of the definition that one unit represents the activity that inhibits cytochrome c reduction by 50%.

### 2.7. Western Blotting

Western blotting (WB) analysis was performed on 40 *μ*g lysates from young and old myoblasts and myotubes, using SOD1 (71G8) mouse mAb (number 4266, Cell Signalling Technology, Danvers, MA, USA) at 1 : 1000, SOD2 (D9V9C) rabbit mAb (number 13194, Cell Signalling Technology) at 1 : 1000, and *β*-actin (8H10D10) mouse mAb (number 3700, Cell Signalling Technology) at 1 : 1000, as primary antibody, and secondary HRP-conjugated antibodies (Cell Signalling Technology) at 1 : 5000. Bands were detected and pictured at Bio-Rad GelDoc by LiteAblot PLUS enhanced chemiluminiscent substrate (EuroClone); densitometry analyses were performed with ImageJ software.

### 2.8. Measurements of Mitochondrial Superoxide Anion Production

Superoxide production was measured using the indicator MitoSOX Red (number M36008, Thermo Fisher Scientific, USA) according to the manufacturer's instructions. Satellite cells were cultured as both myoblasts and myotubes. Before experiments, some of the cultures receive a treatment with 200 *μ*M N-acetyl cysteine (NAC) for 24 hours or with NES containing 10 mM glucose plus 5 mM sodium pyruvate (BURST) solution, as acute stimulus and for 15 minutes and 2 hours. The cells were loaded with 5 *μ*M MitoSOX and incubated for 10 min at 37°C in culture media and washed twice with normal external solution (NES, containing in mM: 140 NaCl, 2.8 KCl, 2 CaCl_2_, 2 MgCl_2_, 10 glucose, 10 N-2-hydroxyethylpiperazine-N′-2-ethanesulfonic acid- (HEPES-) NaOH, pH 7.3). During measurements, samples that received NAC or BURST pretreatment were in NES plus 200 *μ*M NAC or BURST solution. Images were acquired at 512 × 512 pixels using a LSM 510 META system (Zeiss, Jena, Germany) equipped with an Axiovert 200 inverted microscope and a Plan Neofluar oil immersion objective (40x/1.3 NA), and they were pseudocolored in green. In a typical time-series recording, 14 frames were collected at frame rate of 0.1 Hz. MitoSOX fluorescence was acquired by exciting at 543 nm and collecting the emission using an LP filter set to 560 nm. Image analysis was performed by LSM 3.0 software (Zeiss). For each sample, fluorescence images were acquired from five randomly selected fields and off-line analysis of intensity was performed in ROI-representing cell areas, while in time lapse experiment ROIs were selected inside obvious mitochondrial areas. All experiments were performed at room temperature (22–26°C).

### 2.9. Seahorse XF Cell Mito Stress Test

The XF Cell Mito Stress Test was used to measure the oxygen consumption rate (OCR) of myoblasts and myotubes using the Seahorse XFp Extracellular Flux Analyzer (number 102745-100, Seahorse Bioscience, Santa Clara, California). This technique uses specific mitochondrial inhibitors which enable the determination of six parameters for describing the key aspects of mitochondrial function: basal OCR, ATP-linked OCR, spare respiratory capacity (SRC), maximal respiratory capacity, proton leak, and nonmitochondrial respiration. Extracellular flux technology enables rapid, real-time detection of metabolic changes in cellular respiration and glycolysis rate. Cells were seeded in an XFp 8-well cell culture miniplate (Seahorse Bioscience) in three replicates at 25 × 10^4^/well density in 100 *μ*l of growth medium and incubated for 24 hours at 37°C, 5% CO_2_. Before the performance of the Cell Mito Stress Test, some of the cultures received a 200 *μ*M NAC treatment for 24 h. Briefly, myoblasts and myotubes were incubated for 45 min at 37°C in ambient CO_2_ in free-buffered DMEM (number 102353-100, Seahorse Bioscience, Santa Clara, California) (pH = 7.4) containing 1.0 mM sodium pyruvate (number S8636, Sigma-Aldrich), 10 mM glucose, and 2 mM glutamine (number G8540, Sigma-Aldrich). Baseline oxygen consumption (basal OCR) rate and parameters calculated after the addition of each drug were measured 3 times for 3 min each separated by a 3 min mix (18 min total). Following the baseline, 1.6 *μ*M oligomycin (number O4876, Sigma-Aldrich), the ATP synthase inhibitor, was injected into each well, followed by 3 cycles of 3 min mix and 3 min measurement. Then, 0.5 *μ*M of carbonyl cyanide*-*4-(trifluoromethoxy)phenylhydrazone (FCCP, number C2920, Sigma-Aldrich) was injected to uncouple the mitochondrial inner membrane and measure maximal respiration (maximal OCR = maximum electron flux through the electron transfer chain). Lastly, 1 *μ*M antimycin A (number A8674, Sigma-Aldrich) is injected to inhibit electron flux through complex III and so measurement of nonmitochondrial O_2_ consumption. At the end of each experiment, the cells were lysed to determinate the total protein content (BCA assay). The mitochondrial OCR is referred to *μ*g of total cellular proteins and reported as pmol·min^−1^·*μ*g ^−1^.

### 2.10. Seahorse XF Glycolysis Stress Test

In order to quantify changes in the glycolytic function, the glycolysis stress test was performed. It measures the extracellular acidification rate (ECAR, defined as the rate of change in proton excretion from the cell), which reflects the rate of glycolysis. It was carried out under similar conditions as the mitochondrial stress test with some differences. The assay media contained no glucose or pyruvate but supplemented with 2 mM glutamine. After the measurement of the basal ECAR, the cells enter into glycolysis with the injection of 10 mM glucose. Then, 1 *μ*M oligomycin was injected to inhibit OXPHOS and further shift the energy production to glycolysis; the consequent increase in ECAR would reveal the cellular maximum glycolytic capacity. Finally, 50 mM 2-deoxy-glucose (2-DG, number D6134, Sigma-Aldrich) was added; such compound is a glucose analogue that inhibits glycolysis through competitive binding to glucose hexokinase. The resulting decrease in ECAR confirms that the ECAR produced in the experiment is due to glycolysis. The ECAR is referred to *μ*g of total cellular proteins and reported as mpH·min^−1^·*μ*g^−1^.

### 2.11. Statistical Analysis

The Seahorse XF instrument was controlled by Seahorse software for the generation of data files. For data processing, Seahorse Wave software (version 2.3.0) was used. The analysis of OCR and ECAR data was carried out also using the XF Stress Test Report Generator (version 3.0.6). Figure and statistical analyses were generated with the help of GraphPad Prism version 5.0 (GraphPad Software, La Jolla, USA). Results are presented as means ± standard errors (SEM) or as representative traces. The statistical analysis was carried out using the *t* test (^∗^ *p* ≤ 0.05, ^∗∗^ *p* ≤ 0.005, and ^∗∗∗^ *p* ≤ 0.0005). Analysis of mitochondrial fragmentation has been performed using Bonferroni's multiple comparison test. The significance has been codified as ^∗^ *p* ≤ 0.05 and ^∗∗^ *p* ≤ 0.005.

## 3. Results

### 3.1. Characterization and Functionality of Human Myoblast and Myotubes

Human myoblasts from muscle biopsies were isolated, expanded in culture, and characterized by myogenic purity, calculated as the number of cells expressing desmin (% of desmin/^ve+^ cells); ability to fuse, calculated as fusion index (% FI), % of unfused desmin/^ve+^ cells; and population doubling level at day 25 of culture. In Table 1 are summarized the results of myogenic purity and FI percentage for young and elderly cell cultures. Interestingly, even if the number of myogenic cells (desmin/^ve+^ cell %) does not significantly change with the donor's age, the fusion index was significantly lower in primary human elderly myoblasts differentiated for 7 days (resp., 48.2% in young versus 24.3% in elderly). Moreover, as we previously reported [6, 8, 9, 29, 32], we observed a defected regeneration process since from elderly samples myotubes were smaller in number and thinner, containing only few myonuclei (data not shown). Furthermore, the percentage of unfused desmin/^ve+^ cells calculated after 7 days of differentiation increased with age (from 32.6% in young versus 66.8% in elderly), while the myoblasts' proliferative rate (PDL) at 25 days of culture was significantly decreased.

Moreover, for our experiments, we have used myogenic precursors sorted for two specific surface markers: CD56 and 5.1H11. CD56 is a surface marker expressed by human satellite cells either in situ or upon dissociation [33]. 5.1H11 is a specific human myoblast and myotube marker [34]. As it can be seen, in young and old samples, the percentage of desmin/^ve+^ cells (67.5 ± 4.6 and 70.3 ± 8.7, resp.) and CD56/^ve+^/5.1H11/^ve+^ cells 70.1 ± 5.6 and 68.4 ± 4.3, resp.) was not significantly different, confirming a good myogenicity of our cultures and the validity of the choice of characterization markers.

The table summarizes the characterization parameters obtained from young and elderly samples. The percentage of myogenic cells (desmin/^ve+^ cell %) is shown for young and elderly cultures and analyzed by the expression of desmin. To validate whether the expression of desmin was enough and sufficient as myogenic purity index, we further sorted by FACS the primary myoblast for two surface myogenic markers (CD56 and 5.1H11). The fusion index was determined by counting the number of nuclei in differentiated myotubes (positive for MyHC) after 7 days of culture in differentiation medium and reported as a percentage of the total number of nuclei; further, the unfused desmin-positive cells were counted after 7 days of differentiation. Population doubling level calculation at day 25 of culture was used to assess cell proliferative capability. Immunostaining and FI data were derived from at least 1000 cells counted in at least 10 different randomly chosen optical fields of each culture. All data (*n* = 8 young; *n* = 5 old) are presented as mean ± SEM, ^∗^ *p* < 0.05.

**Figure 1:**
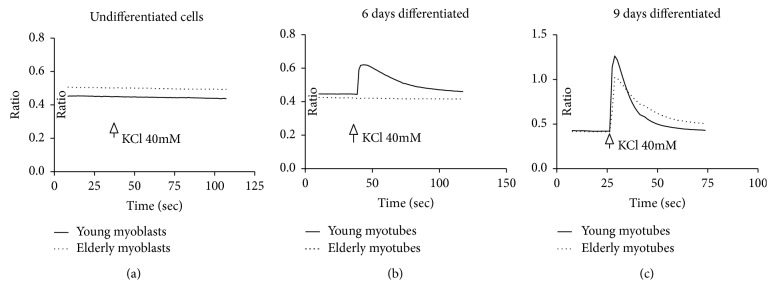
Ca^2+^ imaging and videomicroscopy experiments. Examples of Ca^2+^ transient following depolarization from KCl 40 mM on myoblasts (a) and 6 (b) and 9 (c) in vitro differentiated myotubes obtained from young (solid line) and elderly (dotted line) donors. The figure shows the 340/380 ratio as a function of time (see Methods).

To verify the functionality of the primary myoblast and myotubes used in our experiments, we evaluated the capability to respond at depolarization due to the addition of KCl by generating characteristic Ca^2+^ transients. Here, we assessed the KCl-induced Ca^2+^ elevation in Fura-2-loaded young and elderly myoblasts and myotubes using single cells (*n* = 51 young and *n* = 31 elderly myoblasts; *n* = 26 young and *n* = 47 elderly myotubes after 6 days of differentiation; and *n* = 13 young and *n* = 10 elderly myotubes after 9 days of differentiation). In Figure 1 are reported representative traces of cytosolic Ca^2+^ transients in undifferentiated cells and 6- and 9-day-old differentiated cells. KCl 40 mM caused a transient rise in cytosolic Ca^2+^ levels in myotubes but not in myoblasts as expected (Figure 1(a)). Moreover, 6 days of differentiation were not enough for elderly myotubes to be fully functional since no cytosolic Ca^2+^ rise was observed after KCl addition (Figure 1(b)). Finally, this Ca^2+^ rise was less prominent in elderly myotubes after 9 days of differentiation (Figure 1(c)) with an uncompleted response and delay to return to baseline, conversely in young myotubes.

### 3.2. SOD Antioxidant Enzymatic Activity

We measured the activity of the cytosolic isoform of the antioxidant enzyme SOD1 in cell lysates obtained from myoblast and myotubes both from young and elderly donors. No difference was found comparing young versus old myoblasts or young versus old myotubes. A significant increase in SOD1 activity was observed comparing myoblasts versus myotubes on both young and elderly conditions (^∗^ *p* ≤ 0.05).

### 3.3. Protein Expression of SOD1 and SOD2

**Figure 2:**
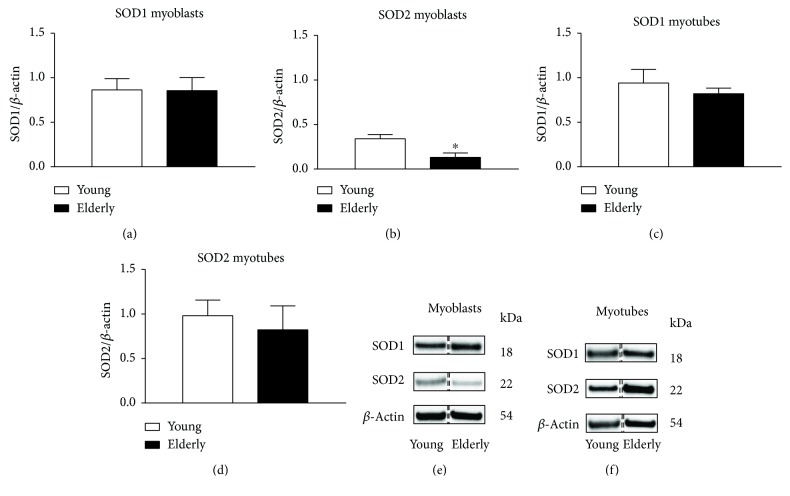
SOD1 and SOD2 protein expression. Western blotting analysis of superoxide enzymes cytosolic type 1 and mitochondrial type 2 was performed on young and elderly myoblasts and myotubes. *β*-Actin content was used for normalization. Panels (a), (b), (c), and (d) show SOD1 and SOD2 densitometric analysis of Western blots performed on three young and three old samples expressed as mean ± SEM. Representative patterns of SOD1, SOD2, and *β*-actin (as loading control) expression in young and elderly myoblasts and myotubes are shown in panels (e) and (f). The bands were taken from two nonadjacent lanes originating from exactly the same gel and blot with exactly the same exposure time, but spliced together indicated with double-dotted lines. Moreover, we performed no change in contrast (^∗^ *p* ≤ 0.05).

We distinguished the two different forms of intracellular SOD, SOD1 and SOD2, cytosolic and mitochondrial protein, respectively. The protein expression was determined on young and elderly samples as both myoblasts and myotubes using Western blot (Figure 2). We observed a statistically significant decrease of SOD2 in elderly myoblasts with respect to young ones (Figure 2(b)). Representative SOD1 and SOD2 bands obtained by myoblasts and myotubes are shown in Figures 2(e) and 2(f), respectively.

### 3.4. Mitochondrial Superoxide Anion Quantification with MitoSOX Red

**Figure 3:**
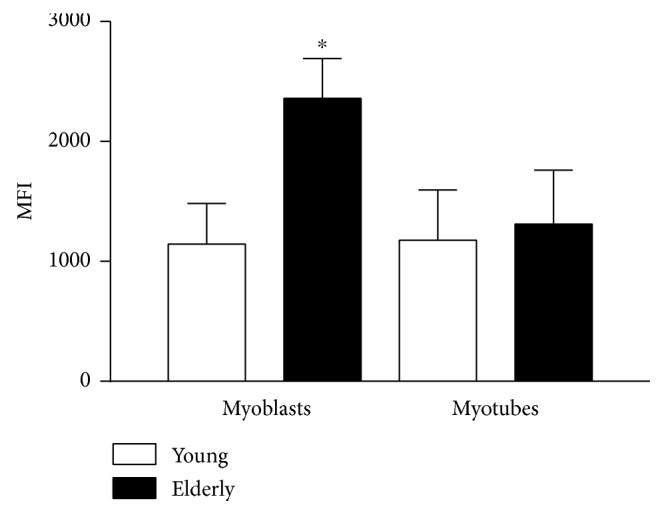
Mitochondrial superoxide anion quantification through MitoSOX Red as basal level. Mitochondrial superoxide anion production was assessed using a MitoSOX Red fluorescent probe. The baseline fluorescence level emitted by MitoSOX when it is oxidized by O_2_^•−^ was measured at the beginning of the time series recordings performed on 3 randomly selected spot on the cells' area. Data are mean fluorescence ± SEM of three independent experiments on elderly samples and of five experiments on young ones (^∗^ *p* ≤ 0.05).

We investigated the production of O_2_^•−^ by the mitochondria in living cells using the MitoSOX Red Indicator. We first determined the mean fluorescence intensity (MFI) of the images at basal condition in untreated control condition at time zero of the time series recordings (Figure 3), and we found that in elderly myoblasts the fluorescence was significantly higher compared to the other samples (^∗^, *p* ≤ 0.05).

**Figure 4:**
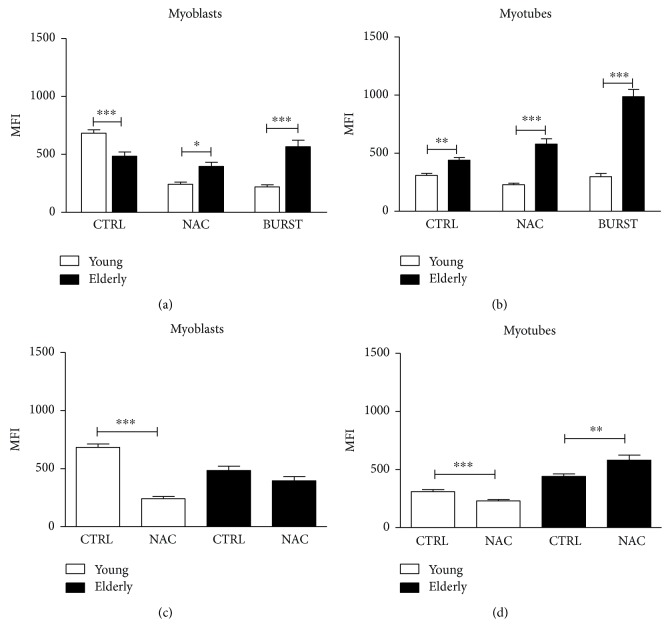
Mitochondrial superoxide anion quantification through MitoSOX Red during time series recordings. Mitochondrial superoxide anion production was assessed using a MitoSOX Red fluorescent probe. Mean fluorescence intensity (MFI) quantitation of confocal images of whole myoblasts (panels a and c) and myotubes (panels b and d) and primary myoblasts as control (CTRL), treated with 200 *μ*M NAC for 24 hours and BURST for 2 hours, were represented. Data are mean ± SEM of three independent experiments on elderly samples and of five experiments on young ones. ^∗^ *p* ≤ 0.05, ^∗∗^ *p* ≤ 0.005, and ^∗∗∗^ *p* ≤ 0.0005.

In addition, myoblasts (Figure 4(a)) or myotubes (Figure 4(b)) of young and elderly subjects were compared for O_2_^•−^ production when treated for 24 hours with 200 *μ*M of the general antioxidant NAC or for 2 hours with high glucose and sodium pyruvate solution to BURST the increase in mitochondrial superoxide.

Elderly myoblasts in the control condition showed a statistically significant decrease in the MFI compared to young samples (Figure 4(a); young *n* = 357, elderly *n* = 111, ^∗∗∗^ *p* ≤ 0.0005). The pretreatment with 200 *μ*M NAC (young *n* = 199, elderly *n* = 111) and BURST (young *n* = 121, elderly *n* = 129) statistically increased the O_2_^•−^ production in the elderly with respect to the young ones (^∗^ *p* ≤ 0.05 in NAC condition and ^∗∗∗^ *p* ≤ 0.0005 in BURST condition). In myotubes (Figure 4(b)), there was a statistically significant increase in mean intensity fluorescence in the elderly with respect to the young samples for all experimental conditions (young CTRL *n* = 229, elderly CTRL *n* = 143, ^∗∗^ *p* ≤ 0.005; young NAC *n* = 187, elderly NAC *n* = 113, ^∗∗∗^ *p* ≤ 0.0005; and young BURST *n* = 137, elderly BURST *n* = 154, ^∗∗∗^ *p* ≤ 0.0005). The MFI of myoblasts (panel c) and myotubes (panel d) are compared to the control and NAC sample conditions. Young myoblasts and myotubes in NAC condition showed a statistically significant decrease in MFI compared to the control condition (^∗∗∗^ *p* ≤ 0.0005). The NAC exposure of elderly myotubes resulted in a statistically significant increase in MFI compared to the control condition (^∗∗^ *p* ≤ 0.005).

### 3.5. Superoxide Anion Coefficient of Variation

**Figure 5:**
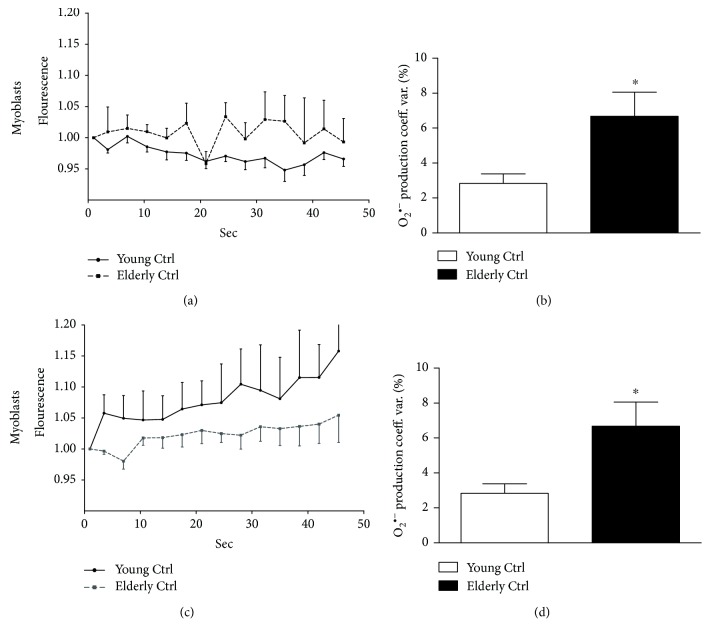
Superoxide anion coefficient of variation. The superoxide anion coefficient of variation was calculated on a short video acquired during the MitoSOX experiment. Traces of the fluorescence registered in 50 seconds of young (panel a) and elderly (panel c) myoblasts and myotubes were represented. The quantification, expressed as percentage of coefficient of variation, was shown in myoblasts (panel b) and in myotubes (panel d). In myoblasts, the O_2_^•−^ coefficient of variation expressed as percentage increased in elderly samples comparing young ones (^∗^ *p* ≤ 0.05) while in myotubes a significant reduction of this parameter was shown in elderly versus young (^∗^ *p* ≤ 0.05).

While performing our MitoSOX analysis, we also acquired short videos recording 14 frames every 3.5 seconds (about 50 seconds) of the MitoSOX signal. We graphed the MFI of young and elderly primary myoblasts versus time (Figure 5) observing that trace's profile differed significantly among samples. To quantify this observation, we used the coefficient of variation (CV), as it is a measure of dispersion of a frequency distribution. This analysis on myoblast-nontreated samples showed that in the elderly, the O_2_^•−^ CV was statistically significantly increased versus young (panel b, ^∗^ *p* ≤ 0.05). On the contrary, elderly myotubes showed a statistically significant reduction of this parameter with respect to the young (panel d, ^∗^ *p* ≤ 0.05).

### 3.6. Mitochondrial Bioenergetics

**Figure 6:**
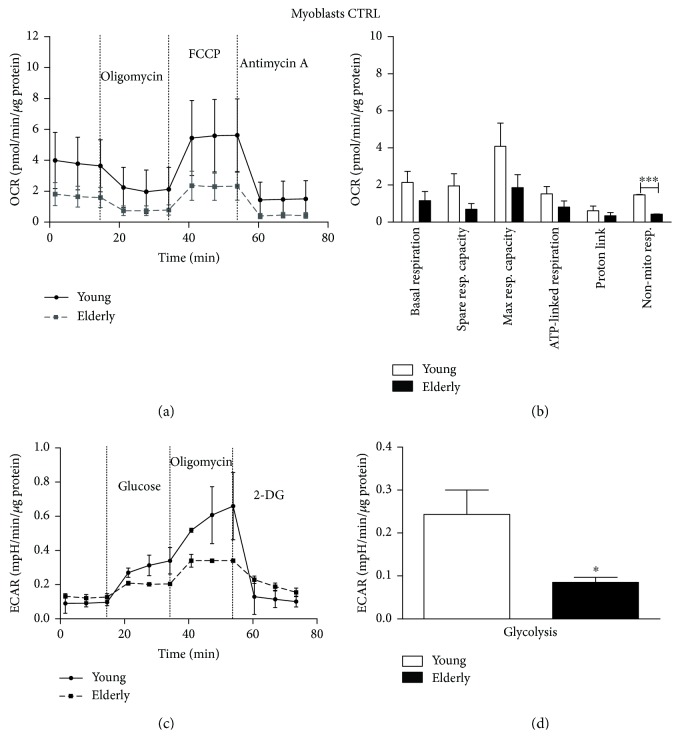
Bioenergetic profiles and parameters in young and elderly myoblasts in the control condition. Mitochondrial respiration (panels a and b) and glycolytic function (panels c and d) of young and elderly myoblasts are represented. OCR parameters were calculated using the data generated in respiratory flux traces (panel a). OCR trace shows basal OCR condition and OCR recordings after 3 sequential additions of, respectively, ATP synthase inhibitor oligomycin, ECT uncoupler FCCP, and complex III inhibitor antimycin A. ECAR trace shows basal ECAR condition and ECAR recordings after 3 sequential additions of, respectively, glucose, oligomycin, and 2-DG. For each sample (*N* = 3 young and *N* = 3 elderly), cells were seeded on an XFp 6-well cell culture miniplate in three replicates at 25 × 10^4^/well density. Results are expressed as mean ± SEM of three independent experiments each performed in triplicate. The OCR and the glycolysis values were normalized to cellular protein content. ^∗^ *p* ≤ 0.05 and ^∗∗∗^ *p* ≤ 0.0005.

**Figure 7 fig7:**
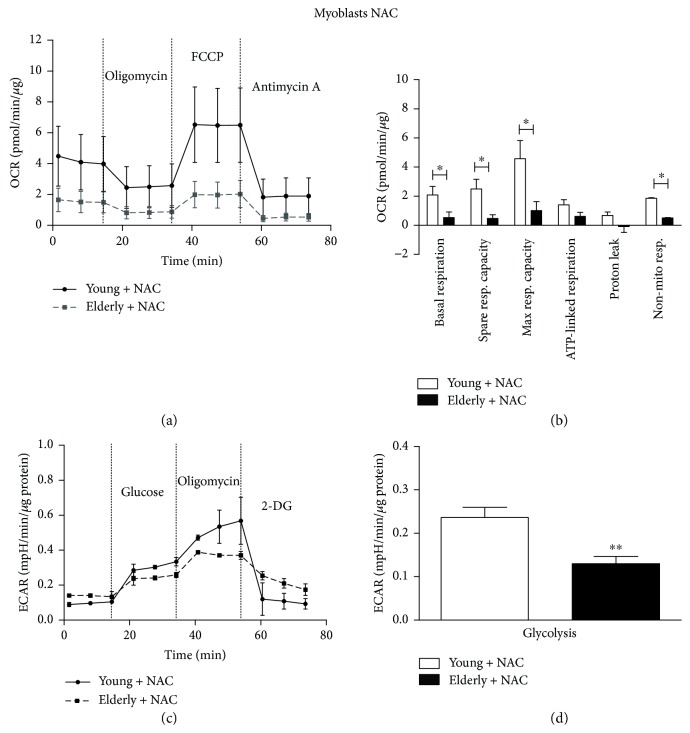
Bioenergetic profiles and parameters in young and elderly myoblasts following NAC exposure. Mitochondrial respiration (panels a and b) and glycolytic function (panels c and d) of young and elderly myoblasts are represented. OCR parameters were calculated using the data generated in respiratory flux traces (panel a). OCR trace shows basal OCR condition and OCR recordings after 3 sequential additions of, respectively, ATP synthase inhibitor oligomycin, ECT uncoupler FCCP, and complex III inhibitor antimycin A. ECAR trace shows basal ECAR condition and ECAR recordings after 3 sequential additions of, respectively, glucose, oligomycin, and 2-DG. For each sample (*N* = 3 young and *N* = 3 elderly), cells were seeded on an XFp 6-well cell culture miniplate in three replicates at 25 × 10^4^/well density. Results are expressed as mean ± SEM of three independent experiments each performed in triplicate. The OCR and the glycolysis values were normalized to cellular protein content. ^∗^ *p* ≤ 0.05 and ^∗∗^ *p* ≤ 0.005.

**Figure 8 fig8:**
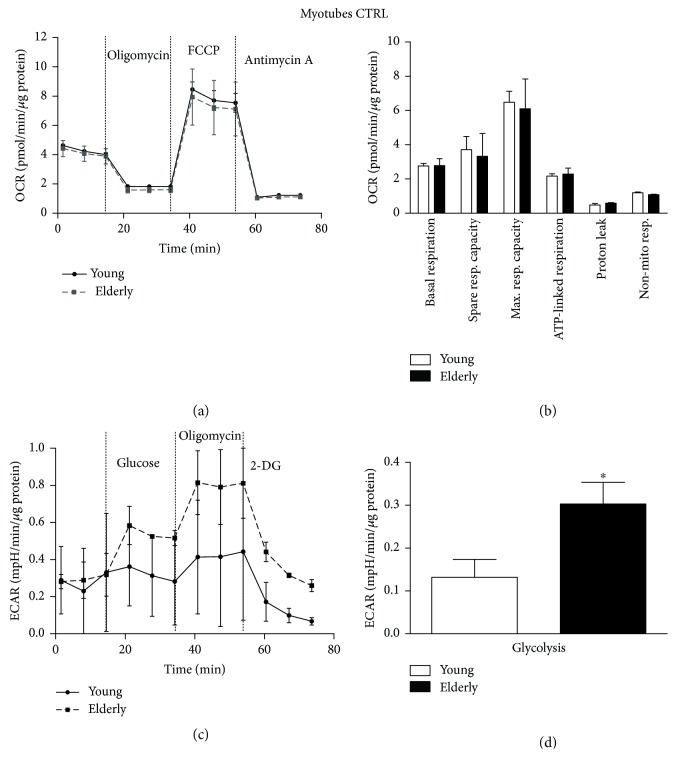
Bioenergetic profiles and parameters in young and elderly myotubes in the control condition. Mitochondrial respiration (panels a and b) and glycolytic function (panels c and d) of young and elderly myotubes are represented. OCR parameters were calculated using the data generated in respiratory flux traces (panel a). OCR trace shows basal OCR condition and OCR recordings after 3 sequential additions of, respectively, ATP synthase inhibitor oligomycin, ECT uncoupler FCCP, and complex III inhibitor antimycin A. ECAR trace shows basal ECAR condition and ECAR recordings after 3 sequential additions of, respectively, glucose, oligomycin, and 2-DG. For each sample (*N* = 3 young and *N* = 3 elderly), cells were seeded on an XFp 6-well cell culture miniplate in three replicates at 25 × 10^4^/well density. Results are expressed as mean ± SEM of three independent experiments each performed in triplicate. The OCR and the glycolysis values were normalized to cellular protein content. ^∗^ *p* ≤ 0.05.

**Figure 9:**
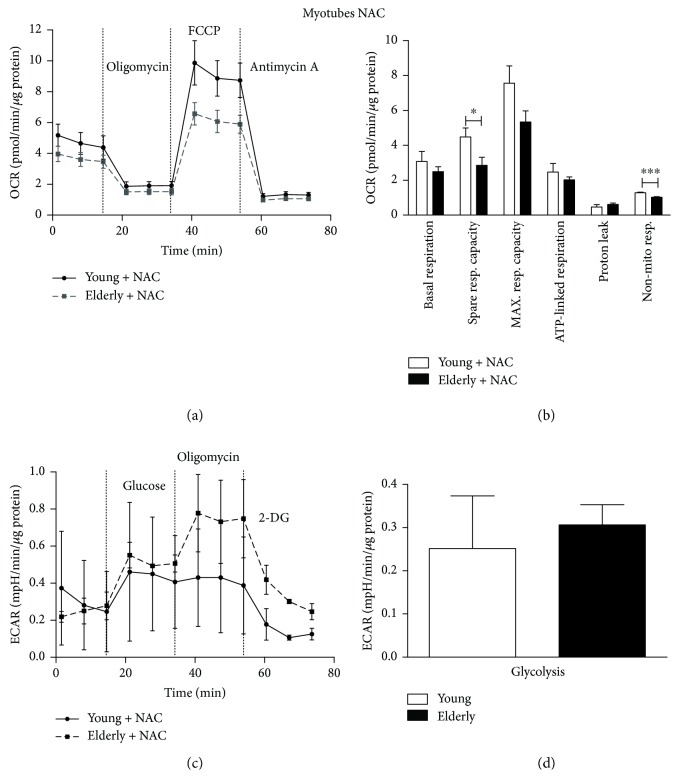
Bioenergetic profiles and parameters in young and elderly myotubes following NAC exposure. Mitochondrial respiration (panels a and b) and glycolytic function (panels c and d) of young and elderly myoblasts are represented. OCR parameters were calculated using the data generated in respiratory flux traces (panel a). OCR trace shows basal OCR condition and OCR recordings after 3 sequential additions of, respectively, ATP synthase inhibitor oligomycin, ECT uncoupler FCCP, and complex III inhibitor antimycin A. ECAR trace shows basal ECAR condition and ECAR recordings after 3 sequential additions of, respectively, glucose, oligomycin, and 2-DG. For each sample (*N* = 3 young and *N* = 3 elderly), cells were seeded on an XFp 6-well cell culture miniplate in three replicates at 25 × 10^4^/well density. Results are expressed as mean ± SEM of three independent experiments each performed in triplicate. The OCR and the glycolysis values were normalized to cellular protein content. ^∗^ *p* ≤ 0.05 and ^∗∗∗^ *p* ≤ 0.0005.

Young and elderly samples (either myoblasts or myotubes) were assayed in untreated control condition and 24 hours after NAC treatment (Figures 6 [Fig fig7] [Fig fig8]–9). In the control condition (Figure 6), the OCR parameters showed a decreasing trend in elderly versus young. However, only the nonmitochondrial respiration was significantly lower in the elderly (^∗∗∗^ *p* ≤ 0.0005). Concerning the ECAR profile, the glycolysis parameter is a measure of extracellular acidification reached by the cells following the addition of saturating amounts of glucose. We found that glycolysis was significantly lower in the elderly compared to the young (^∗^ *p* ≤ 0.05).

Following NAC exposure (Figure 7), the differences between elderly and young became statistically significant in most of OCR parameters, such as the basal respiration, the spare respiratory capacity, and the maximal respiratory capacity (^∗^ *p* ≤ 0.05), suggesting that NAC is more effective in scavenging ROS in the young than in the elderly. The glycolysis parameter, obtained by the ECAR profile, was found significantly decreased in elderly myoblasts compared to young ones (^∗∗^ *p* ≤ 0.005).

Other glycolytic parameters (glycolytic capacity, glycolytic reserve, and nonglycolytic acidification, not shown) were not statistically different comparing young versus elderly myoblasts.

After 7 days of differentiation (Figure 8), we did not observe statistically significant differences in OCR parameters in our control condition samples. Surprisingly, the glycolysis was statistically significant higher in elderly myotubes compared to young ones (^∗^ *p* ≤ 0.05).

After NAC treatment (Figure 9), the spare respiratory capacity and nonmitochondrial respiration were significantly decreased (^∗^ *p* ≤ 0.05 and ^∗∗∗^ *p* ≤ 0.0005, resp.) in elderly versus young ones, suggesting that the scavenging activity of the antioxidant may be more considerable on the young than on the elderly. After NAC exposure, glycolysis value did not change comparing young and elderly myotubes.

Similarly to the values obtained for myoblasts, also in myotubes other glycolytic parameters (glycolytic capacity, glycolytic reserve, and nonglycolytic acidification, not shown) were not statistically different comparing young versus elderly.

## 4. Discussion

Aging is known to be characterized by an increase in oxidative stress related to an imbalance between ROS production, in particular O_2_^•−^, and scavenger activity of antioxidant enzymes [35, 36]. This altered oxidative management is also reflected in the regenerative capacity of the skeletal muscle of the elderly, which shows an impairment in the primary myoblast-differentiating process responsible for regeneration [9, 29]. The characterization of our primary myoblast populations shows in the elderly a less regenerative capability with respect to the young ones, demonstrated by a slower proliferative rate and difficulty in differentiation (reduced % of FI and increased % of unfused desmin/^ve+^ cells). The difficulty in functional differentiation, which we already previously demonstrated [29, 37], is confirmed by the response at depolarization stimuli (40 mM KCl) of the elderly myotubes that it is not yet complete and delays to return to baseline (Figure 1).

The O_2_^•−^ radical, which we had already shown to be higher in the elderly's whole myoblasts [8], could therefore represent, if not adequately contrasted, a potential harmful agent. On the other hand, a transient increase in O_2_^•−^, readily reduced by scavenger enzymes, seems to be positively correlated with a sustained metabolism, as occurs during the normal mitotic process or during the early phase of regeneration [38], whereas a subsequent switch-off may accelerate the muscle differentiation [39]. On the contrary, its prolonged presence, due to poor or insufficient antioxidant activity, can cause damage and impair the differentiation process.

**Figure 10:**
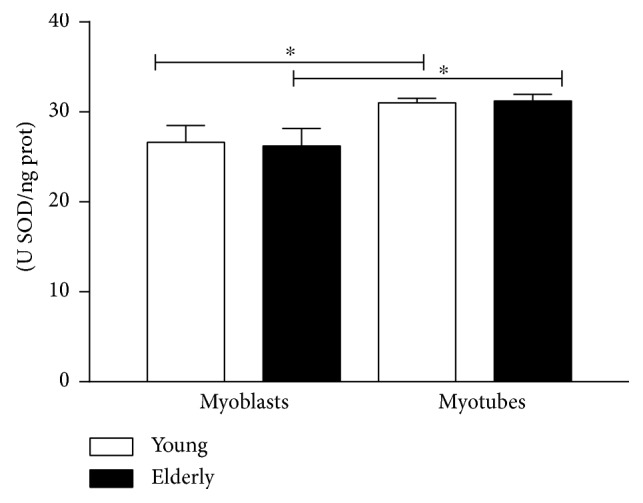
Superoxide dismutase activity. Quantitative analyses from young and elderly myoblasts and myotubes for the activity of the superoxide dismutase enzyme, SOD1. The SOD units (U SOD) were calculated considering that 1 SOD unit is defined as the quantity that inhibits the rate of cytochrome c reduction by 50% per ng of protein. The activity of the superoxide dismutase was expressed as U SOD/ng protein. ^∗^ *p* ≤ 0.05.

In this study, we analyzed the activity of SOD1, noting that the differentiation process per se increases SOD1 activity independently of the donor age (Figure 10). On the other hand, we have highlighted a higher basal level of O_2_^•−^ in the mitochondria of elderly myoblasts (Figure 3) that correlates with a low expression of SOD2. These data lead us to hypothesize that the highest O_2_^•−^ levels observed in the whole myoblasts may be due to a mitochondrial production not sufficiently contrasted by SOD2 (Figures 2(a) versus 2(b)), thus preventing its switch-off useful for differentiation. Consistent with this, other studies show that a complete differentiation process occurs only in young samples, while it is impaired in elderly ones [8, 9]. The unexpected high mitochondrial O_2_^•−^ level found in young myoblasts could be explained considering that they are characterized by an increased rate of proliferation [32] together with a higher oxygen consumption rate with respect to elderly ones [40], as we demonstrated in the present study through bioenergetics analysis (Figure 6). Conversely, the lower O_2_^•−^ levels found in young myotubes compared to myoblasts could be justified by the increase in SOD activity and the lack of proliferation rate effect as we are considering the differentiation phenotype.

As antioxidant and oxidant agents can affect the O_2_^•−^ production and thus activate its scavenging by SOD enzyme, we stimulated our myoblasts and myotubes with NAC and BURST solution, respectively.

Elderly myoblasts and myotubes (Figures 4(c) and 4(d)) did not show lower O_2_^•−^ levels after the NAC treatment, while young myoblasts and myotubes properly responded to NAC exposure by significantly reducing the production of O_2_^•−^ (^∗∗∗^ *p* ≤ 0.0005). These results suggest that first, an increase in O_2_^•−^ production might be not associated with a proportional increase in SOD, and second, solely in samples from young donors might NAC be able to promote cell fitness and viability, since elderly cells even showed significantly higher O_2_^•−^ levels in the presence of NAC compared to the control condition (^∗∗^ *p* ≤ 0.005).

In aged muscles [35] as well as in primary myoblasts [9, 29], the impairment of the antioxidant machinery was already demonstrated. Therefore, when elderly primary myoblasts are treated with a high glucose solution (BURST) that enhances their cellular metabolism, they will uncontrollably reach the highest levels of O_2_^•−^ production Consistently, treatment with an antioxidant agent like NAC, which acts as glutathione precursor [41, 42], might not significantly affect elderly primary myoblasts due to the impaired activity of glutathione-dependent enzymes and the accumulation of hydrogen peroxide [9]. Furthermore, short videos acquired during MitoSOX experiments allowed us to investigate the variation of the response in O_2_^•−^ production expressed as coefficient of variation (CV) in young and elderly samples under control conditions. The CV was found higher in elderly myoblasts with respect to young myoblasts, while an opposite result was observed in myotubes. We can argue that the increased variability in O_2_^•−^ production in elderly myoblasts could be due to a large dispersion in the frequency distribution of O_2_^•−^ flickering or, in other words, we can imagine that the elderly mitochondria produce O_2_^•−^ in a slow and continuous regimen with respect to young mitochondria. In elderly myotubes, the O_2_^•−^ flickering has a narrow frequency distribution, significantly reduced with respect to the young ones, probably because those rely more on glycolysis than mitochondrial OXPHOS.

ROS not only have a detrimental effect on biological molecules, such as DNA, proteins, and lipids [36], due to their accumulation but also affect myogenic regeneration at several stages. Specifically, ROS enhanced NF-*κ*B transcription factor-activating I*κ*B kinase (IKK) that phosphorylates the I*κ*B inhibitor in turn upregulating NF-*κ*B [39]. In the skeletal muscle, the role of this transcription factor is related to the activation of protein degradation pathways [43]. Consequently, we can argue that a high level of O_2_^•−^ found in our elderly myotubes with respect to young ones (^∗∗^ *p* ≤ 0.005) could result in the activation of NF-*κ*B that in turn could boost cell catabolism. Consistent with this, we and other groups previously demonstrated that elderly samples' upregulation of FOXO1A, myostatin genes, and NF-*κ*B protein expression, together with downregulation of AKT1/PIK3CA/MTOR genes and phospho-Akt protein expression, could lead to the activation of the atrophic/catabolic pathway at the expense of the anabolic ones and finally inhibit muscle differentiation [8, 9, 32]. Overall, our data, along with the results of previous studies, suggest that the failure to differentiate *in vitro* and to regenerate muscle *in vivo* [9, 10] could be associated with the oxidative damage accumulation, impaired antioxidant activity, and insufficient repair capability observed in the elderly. As all the above-mentioned events take place in mitochondria, a brief video of MitoSOX experiments performed on primary myoblasts from young or elderly donors was inserted in the manuscript (link to movies here as supplementary material; young and elderly myoblasts) to show their relative mitochondrial dynamic.

Furthermore, to deeply investigate mitochondria's functions, we also analyzed mitochondrial bioenergetics in our samples via the Seahorse Flux analyzer. This instrument monitors OXPHOS by measuring in real time OCR and glycolysis by measuring in real time ECAR. Revising literature on aged muscle bioenergetics, many studies demonstrated a significant decrease in the activity of several respiratory chain complexes and a substantial increase in ROS production, thus confirming the mitochondrial theory of aging [44, 45]. Measurements of mitochondrial respiration are strong indicators of the functional bioenergetic capacity of mitochondria and of overall cellular health. The Seahorse Mito Stress Test uses modulators of cellular respiration that specifically target components of the electron transport chain to reveal key parameters of metabolic function. The first compound injected is oligomycin that inhibits ATP synthase. The second compound is FCCP, an uncoupling agent that permeabilizes the inner mitochondrial membrane to protons, forces the mitochondria to increase the flow of electrons (and thus oxygen consumption) to maintain the membrane potential. As a result, electron flow through the ETC is constant and oxygen is maximally consumed by complex IV. The last drug injected is antimycin A, a complex III inhibitor that shuts down the mitochondrial respiration. Parameters from the cellular mitochondrial function assay give insights into different aspects of mitochondrial functions.

In myoblasts, we observed a decreasing trend for many OCR parameters when elderly and young samples were compared, especially considering the nonmitochondrial respiration (Figures 6(a) and 6(b)). Notably, the lower values for spare respiratory and maximal respiratory capacity, which are considered as indicators of metabolic plasticity, would indicate compromised mitochondrial mass or integrity [46] and defective mitochondria in elderly samples. Moreover, we found that the glycolytic rate was significantly lower in elderly compared to young samples (Figures 6(c) and 6(d)).

After the NAC exposure, we observed that basal respiration, spare respiratory capacity, maximal respiration capacity, and nonmitochondrial respiration became significantly lower in elderly myoblasts versus young ones (Figures 7(a) and 7(b)), together with a more pronounced decrease in glycolysis (Figures 7(c) and 7(d)). As highlighted before, NAC is solely effective in scavenging O_2_^•−^ produced by young myoblasts (Figure 4(c)); therefore, the exclusive activity of NAC on young myoblasts could lead to a boost in their viability whose occurrence would justify their improved mitochondrial performance. On the contrary, we observed no differences between untreated and NAC-treated elderly myoblast in terms of OCR parameters, in agreement with the comparable O_2_^•−^ levels detected for both conditions (Figure 4(c)), due to a possibly partially compromised antioxidant machinery. This may lead to the hypothesis that the lower OXPHOS which we see in myoblasts associated to a whole decreased glycolysis could result in a less aerobic glycolitic component, leaving perhaps the role of energy production almost exclusively to the anaerobic component.

This hypothesis would be in agreement with the recent study conducted by Pääsuke et al. [40] on primary myoblasts from young and elderly subjects demonstrating that the proliferation of myoblasts *in vitro* (from passages 2 to 6, simulating an *in vitro* senescence) is associated with downregulation of OXPHOS, and aging *in vivo* (young versus elderly) caused an altered metabolic profile by favoring the glycolytic pathway. Our results are in agreement also with another recent study demonstrating that senescent myoblasts show a metabolic shift leading to glycolytic enzymes a pronounced downregulation in glycolysis [47].

In myotubes, we found a similar trend for the OCR parameters of elderly versus young donors. The myogenic differentiation process is usually accompanied by an increase in oxidative metabolism with a shift from glycolysis to OXPHOS, as the major energy demand occurs [38, 48]. This phenomenon is not observed in our samples, where elderly myotubes have an OXPHOS metabolism trend similar to young ones, but a significantly increased glycolytic rate. These findings could suggest a different energetic plasticity of elderly myotubes with respect to young ones and the increased energy demand could not be covered only by OXPHOS metabolism but require an incremented glycolytic process maybe because their mitochondrial machine is subcritically impaired. Accordingly, Korolchuk and collaborators demonstrated that in senescent cells, the fraction of ATP produced by OXPHOS decreases, while relatively more ATP is generated by anaerobic glycolysis as a compensatory response to mitochondrial dysfunction. Furthermore, early occurrence of mitochondrial dysfunction during the induction of cell senescence could set off a number of different cellular responses and signaling pathways as well as reducing capacity to respond to peak energy demands [49]. Similarly, to myoblasts, NAC treatment disclosed the real differences in few of the measured mitochondrial parameters (e.g., spare respiratory capacity and nonmitochondrial respiration) between elderly versus young myotubes, confirming the hypothesis that the antioxidant acts by improving the whole physiological fitness of young cells.

## 5. Conclusions

The aging process produces increased mitochondrial O_2_^•−^ levels, neither contrasted by an adequate amount of SOD2 nor scavenged by a NAC or BURST stressor. The proliferative state shows a slower metabolism characterized by less OXPHOS and glycolysis, probably to the detriment of the aerobic glycolytic component.

The metabolic burst due to the onset of differentiation leads to a pronounced increase in glycolysis which does not occur in young myotubes. This result suggests that OXPHOS in elderly myotubes may not be as effective at producing ATP as it is in young myotubes. The increased glycolytic rate observed in elderly myotubes could be therefore considered as part of a compensatory mechanism mounted by the aging cells in response to the sudden increase in energy demand and in the absence of an extra OXPHOS support.

The data collected in the present study lead us to conclude that the elderly may have less metabolic plasticity due to the impaired mitochondrial function caused by oxidative stress. Further studies on the expression and activity of mitochondrial complexes in myotubes with respect to myoblasts could lay the foundations for future investigations about the detailed causes of impaired muscle regeneration during aging.

## Figures and Tables

**Table 1:** Myogenicity, fusion index, unfused desmin/^ve+^ percentage, and proliferation rate (PDL).

Samples	Desmin^/ve+^ cell %	CD56^+^/5.1H11^+^ cell %	Fusion index %	Unfused desmin^/ve+^ cell %	PDL at 25 days of culture
Young	67.5 ± 4.6	70.1 ± 5.6	48.2 ± 4.7	32.6 ± 6.1	13.2 ± 1.2
Elderly	70.3 ± 8.7	68.4 ± 4.3	24.3 ± 6.3^∗^	66.8 ± 13.6^∗^	9.0 ± 0.4^∗^

## Data Availability

The data related to “elderly sample myotubes were smaller in number and thinner, containing only few myonuclei (data not shown)” used to support the findings of this study are available from the corresponding author upon request.

## Abbreviations

ROS: Reactive oxygen species
O2: Superoxide anion
SOD: Superoxide dismutase
OXPHOS: Oxidative phosphorylation.

## Supplementary Materials

Supplementary Materials Video: in the first part of the video, group A includes myoblasts of the young control sample showing the mitochondrial dynamics during a brief recording; in the second one, we can appreciate group B which includes myoblasts of the elderly control sample showing the mitochondrial dynamics during a brief recording. The video shows the different mitochondrial dynamics, with a less activity in the elderly sample with respect to young ones, confirming a reduction of plasticity and metabolism of the elderly. The videos were acquired recording 14 frames every 3.5 seconds (for about 50 seconds). The recordings are compressed 1 : 10 with Zeiss LSM 3.0 SP3 software.
