# Corrigendum to “Superoxide Anion Production and Bioenergetic Profile in Young and Elderly Human Primary Myoblasts”

**DOI:** 10.1155/2021/9764701

**Published:** 2021-04-02

**Authors:** Mariangela Marrone, Rita Maria Laura La Rovere, Simone Guarnieri, Ester Sara Di Filippo, Giovanni Monaco, Tiziana Pietrangelo, Geert Bultynck, Stefania Fulle, Rosa Mancinelli

**Affiliations:** ^1^Department of Neuroscience Imaging and Clinical Sciences, University “G. d'Annunzio” Chieti-Pescara, Via dei Vestini 29, 66100 Chieti, Italy; ^2^Interuniversity Institute of Myology (IIM), Chieti, Italy; ^3^Laboratory of Molecular and Cellular Signaling, KU Leuven, Campus Gasthuisberg O/N-I bus 802, Herestraat 49, 3000 Leuven, Belgium

In the article titled “Superoxide Anion Production and Bioenergetic Profile in Young and Elderly Human Primary Myoblasts” [1], there was a figure enumeration error where Figure 10 should have been placed as Figure 2. Consequently, Figure 10 is not positioned near the part of the text in which it is discussed. Therefore, in Results, the sentence “A significant increase in SOD1 activity was observed comparing myoblasts versus myotubes on both young and elderly conditions (^∗^*p* ≤ 0.05).” is corrected to “A significant increase in SOD1 activity was observed comparing myoblasts versus myotubes on both young and elderly conditions (Figure 10, ^∗^*p* ≤ 0.05).”

Also, there were some errors found in Figure 1. In (c), the *y*-axis legend should read “Myotubes” instead of “Myoblasts.” (d) is the copy of (b). The corrected figure is shown below:

## Figures and Tables

**Figure 1:**
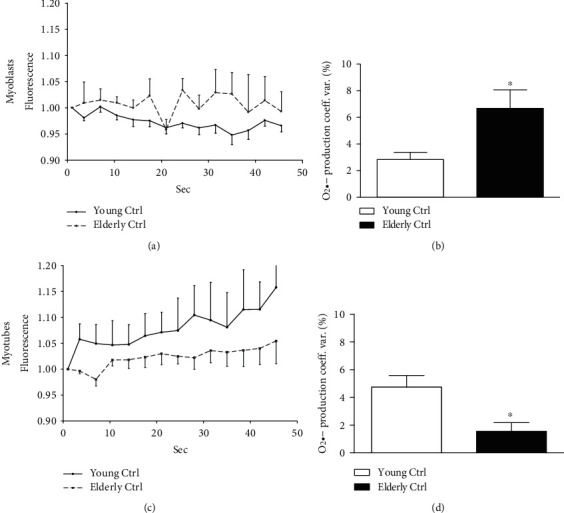
Superoxide anion coefficient of variation. The superoxide anion coefficient of variation was calculated on a short video acquired during the MitoSOX experiment. Traces of the fluorescence registered in 50 seconds of young (a) and elderly (c) myoblasts and myotubes were represented. The quantification, expressed as percentage of coefficient of variation was shown in myoblasts (b) and in myotubes (d). In myoblasts, the O_2_•− coefficient of variation expressed as percentage increased in elderly samples comparing young ones (^∗^*p* ≤ 0.05), while in myotubes, a significant reduction of this parameter was shown in elderly versus young (^∗^*p* ≤ 0.05).
